# Crystal structure of cGMP-dependent protein kinase reveals novel site of interchain communication

**DOI:** 10.1186/1471-2210-11-S1-O13

**Published:** 2011-08-01

**Authors:** Wolfgang R Dostmann, Brent W Osborne

**Affiliations:** 1 Department of Pharmacology, College of Medicine, University of Vermont, Burlington, VT 05405, USA

## Background

The cGMP-dependent protein kinase (PKG) is widely expressed in mammalian tissues and serves as an integral component of second messenger signaling in a number of biological contexts including vasodilation, motility and memory [1]. PKG assembles into homodimers [2] and large conformational changes are induced by the cooperative binding of cGMP [2-4]. However, the structure of PKG and the molecular mechanisms associated with protomer communication following cGMP binding remain obscure.

## Results

Here we report the 2.5 Å crystal structure of a regulatory domain fragment containing both tandem cGMP binding sites of PKG Iα (amino acids 78-355). The overall domain topology of this structure shows a distinct and segregated architecture. The cGMP binding sites are separated by an extended central helix and offer limited interdomain communication. However, a previously uncharacterized helical domain (switch helix) promotes the assembly of a dimeric interface between PKG Iα78-355 protomers. Disruption of this interface by alanine scanning mutagenesis in full length PKG resulted in a marked reduction of activation constants.

## Conclusion

Our results offer new insight about PKG holoenzyme assembly as they provide the first detailed molecular view of tandem cGMP-binding domains and characterize the switch helix as a critical site of interchain communication between PKG protomers. The biological integrity of PKG appears to be mediated by this interface as it is necessary for the maintenance of kinetic fidelity. This structure highlights the critical importance of dimer communication in PKG biology and will likely serve as an improved platform for the strategic development of therapeutic agents aimed at treatment and prevention of cGMP-dependent pathologies. 
